# Advancements in ovarian cancer immunodiagnostics and therapeutics via phage display technology

**DOI:** 10.3389/fimmu.2024.1402862

**Published:** 2024-05-28

**Authors:** Yang Li, Xiao-meng Li, Kai-di Yang, Wei-hua Tong

**Affiliations:** ^1^ Obstetrics and Gynecology Center, First Hospital of Jilin University, Changchun, Jilin, China; ^2^ Department of Rehabilitation, School of Nursing, Jilin University, Changchun, China

**Keywords:** ovarian cancer, phage display technology, targeted drug delivery, immunodiagnostics, personalized therapeutic strategies

## Abstract

Ovarian cancer, ranking as the seventh most prevalent malignancy among women globally, faces significant challenges in diagnosis and therapeutic intervention. The difficulties in early detection are amplified by the limitations and inefficacies inherent in current screening methodologies, highlighting a pressing need for more efficacious diagnostic and treatment strategies. Phage display technology emerges as a pivotal innovation in this context, utilizing extensive phage-peptide libraries to identify ligands with specificity for cancer cell markers, thus enabling precision-targeted therapeutic strategies. This technology promises a paradigm shift in ovarian cancer management, concentrating on targeted drug delivery systems to improve treatment accuracy and efficacy while minimizing adverse effects. Through a meticulous review, this paper evaluates the revolutionary potential of phage display in enhancing ovarian cancer therapy, representing a significant advancement in combating this challenging disease. Phage display technology is heralded as an essential instrument for developing effective immunodiagnostic and therapeutic approaches in ovarian cancer, facilitating early detection, precision-targeted medication, and the implementation of customized treatment plans.

## 1 Introduction

Ovarian cancer is identified as the seventh most common malignancy affecting women across the globe (1). Its significance is highlighted by GLOBOCAN estimates, which report that in 2020, approximately 314,000 women were diagnosed with ovarian cancer, leading to 207,000 fatalities. This positions ovarian cancer as the eighth leading cause of cancer-related incidence and mortality among women internationally (2). Forecasts for the year 2040, based on the assumption that incidence rates remain constant at the 2020 levels, predict around 428,000 new ovarian cancer cases and 307,000 fatalities annually. Achieving a 30% reduction in these figures by 2040 necessitates an annual decrease of 2% in the global incidence and mortality rates of this disease (3). The urgent need for the development of efficacious treatments for gynecological cancers, particularly ovarian cancer, which is known as the deadliest gynecological malignancy, continues to be a significant clinical challenge. This is largely attributed to the subtle, non-specific symptoms of ovarian cancer and the commonality of late-stage diagnoses (4). The complexity of ovarian cancer diagnosis and treatment has ignited intensive efforts to develop screening methodologies that enable early detection in asymptomatic individuals. Despite this, the effectiveness of current leading methods, including CA125 blood tests and transvaginal ultrasound (TVS), has not been conclusively shown to lower ovarian cancer mortality rates in the general population through randomized controlled trials (5). This urgency is further intensified by the challenges presented by advanced, recurrent, and drug-resistant forms of ovarian cancer, highlighting an acute need for novel therapeutic solutions (5). Consequently, this multifaceted dilemma has motivated the research community to push the boundaries of ovarian cancer research, seeking innovative solutions informed by a comprehensive understanding of the intricate dynamics of cancer progression (6). In this context, the advent of targeted diagnostic and therapeutic strategies emerges as a promising development, potentially heralding significant advancements in the management of ovarian cancer. Particularly in the domain of ovarian cancer therapeutics, there has been a notable increase in the development of targeted agents, often aimed at “shared antigens” found across a range of tumors (7). However, a detailed analysis suggests a complex scenario where the effectiveness of these agents may be limited in eliciting strong immune responses due to the ubiquitous nature of these “shared antigens” across various body tissues. This complexity introduces a risk of unintended autoimmune reactions and could contribute to the development of drug resistance (7). As research delves into the nuanced interaction between targeted therapies and the immune microenvironment, it becomes clear that customizing precision medicine strategies to minimize off-target effects and immune-related adverse events is crucial for improving the therapeutic outcomes of these interventions. This approach underscores the importance of advancing targeted treatments within a framework that carefully considers the broader implications on the immune system and patient health.

In the landscape of oncology research, phage display technology has recently stood out as a significant breakthrough, particularly in the realms of immunodiagnostics and therapeutic development for ovarian cancer. This method allows for the comprehensive screening of extensive phage-peptide libraries, facilitating the identification of ligands that bind with high specificity and affinity to cancerous cells, thus enabling precise targeting strategies (8). Such an approach promises to enhance the accuracy of drug targeting, reduce off-target effects, and minimize associated adverse events. Nonetheless, the path to fully leveraging this technology is fraught with obstacles. Phage-peptide display presents short peptides via bacteriophages, screening large libraries efficiently but with limited binding affinity and specificity. Nanobodies from camelids offer high specificity and stability on phages, though they face production challenges. Single-chain variable fragments (scFvs) provide smaller, tissue-penetrating antibody forms with potential stability issues. Fragment antigen-binding (Fab) displays, incorporating complete antibody domains, ensure high stability and affinity but may incur higher costs and reduced tissue penetration. The quest to discover novel and effective phage-peptides is a demanding endeavor that requires a detailed and laborious process. This journey involves repeated cycles of screening and sequencing against tumor cells or tissues, along with stringent validation of ligand binding affinities. Achieving success in this domain necessitates the synthesis of phage-peptides, which are integral to the development of precision-targeted cancer therapeutics (9). This review is dedicated to a comprehensive examination and integration of the existing literature on the application of phage display technology in the immunodiagnostics and treatment of ovarian cancer. Its goal is to delineate the significant progress made, highlight existing knowledge gaps, and outline prospective research trajectories within this area. By doing so, it seeks to furnish insights that are of critical relevance to both the scientific community and clinical practitioners.

## 2 Applications of phage display in cancer research

### 2.1 Phage display vectors: diversity and applications

**Figure 1:**
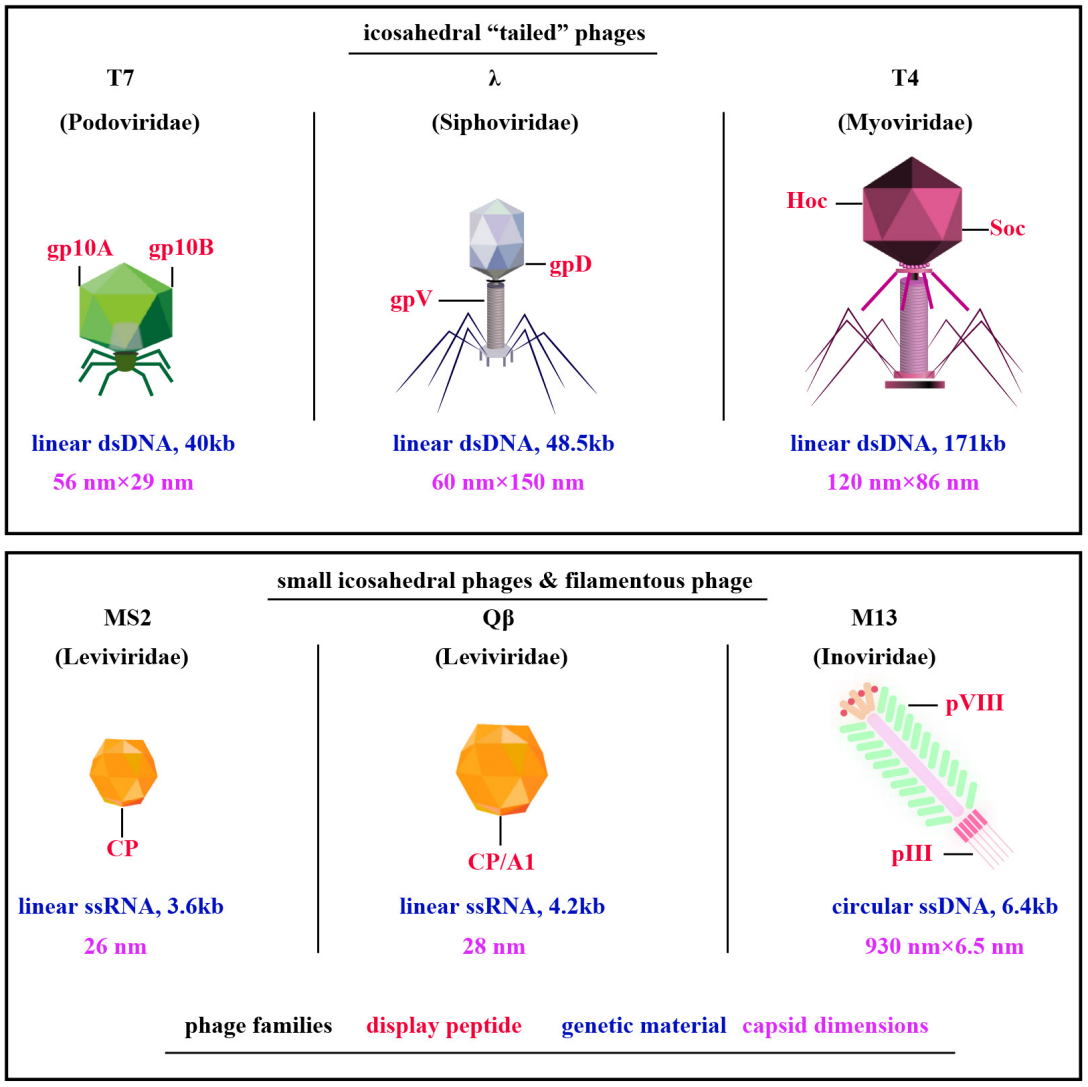
Diverse Phage Types Exploited in Phage Display. Among them, filamentous M13 phages are prominent, along with “tail” phages like T7, λ, and T4, and compact ssRNA icosahedral phages MS2 and Qβ. Notably, key capsid proteins for peptides display are highlighted in red, phage families in black, genetic material in blue, and capsid dimensions in purple [adapted from (11)].

Exploration of phage display technologies has unveiled a plethora of vector systems, including but not limited to M13, T4, T7, λ, Qβ, and MS2 phages (10) (**Figure 1**). The M13 phage, a filamentous non-lytic virus, excels in phage display due to its unique structure and compatibility with Escherichia coli, enabling the presentation of diverse peptides and proteins (12). T4 bacteriophage, lytic with a slender icosahedral head and complex tail, is essential for vaccine delivery, expressing coat proteins like gp23, gp24, gp20, Soc, and Hoc that aid DNA encapsulation and antigen presentation (13, 14). T7, a lytic Podoviridae member, efficiently displays large proteins thanks to its 60 nm head and 40kb DNA, with proteins like gp10A and gp10B enhancing secretion and replication (15–17). The Qβ phage, a minimalist Leviviridae member, features a simple 28nm diameter and a 4.2 kb ssRNA genome that encodes key proteins such as CP, A2, and A1, critical for infecting Escherichia coli and display technologies (18, 19). Similarly, the MS2 phage, also from the Leviviridae family, presents a lytic 26 nm icosahedral structure with a 3.6 kb ssRNA genome coding for proteins like CP, A, replicase, and lysis protein L, essential for assembly and display applications (18, 19). M13, T4, T7, λ, Qβ, and MS2 phages are tailored for distinct biotechnological uses (**Table 1**). M13 facilitates phage display for peptide and protein engineering through non-lytic DNA packaging, supporting drug development and gene therapy. T4 combats antibiotic-resistant bacteria and aids molecular biology research. T7, known for its strong promoter, is essential for high-level protein expression and CRISPR applications. Lambda phage plays a critical role in molecular cloning and E. coli lysis, enhancing phage therapy. Qβ contributes to vaccine development and RNA research, while MS2 is vital in RNA studies and virology education due to its straightforward RNA genome. M13’s non-lytic replication enables the continuous production of large peptide or protein libraries.

**Table 1:** The pros and cons of distinct phage display vectors.

Phage Vector	Pros	Cons
M13	High-level peptide display	Limited to filamentous phage biology
	Non-lytic, continuous display	May require specialized host strains
	Useful for panning and affinity maturation	
T4	High payload capacity	Lytic cycle can complicate library maintenance
	Robust, large capsid for display	Less common for peptide display
	Effective for vaccine development	
T7	Extremely efficient expression	Lytic nature requires careful handling
	Rapid lytic cycle speeds up experiments	Smaller display capacity compared to T4
	Suitable for high-throughput screening	
λ (Lambda)	High DNA packaging efficiency	Complex lifecycle can be a challenge
	Allows for large DNA insertions	Lytic and lysogenic cycles
	Well-studied genetics and molecular biology	
Qβ	RNA phage, useful for RNA display	RNA genome stability issues
	Can display peptides and proteins	Limited host range
	Useful for vaccine development	
MS2	Small RNA phage, simple genome	Limited display capacity
	Useful for studying RNA-protein interactions	Stability and robustness issues
	Potential applications in targeting RNA	

### 2.2 Phage display for cancer immunodiagnostics and therapy

**Figure 2:**
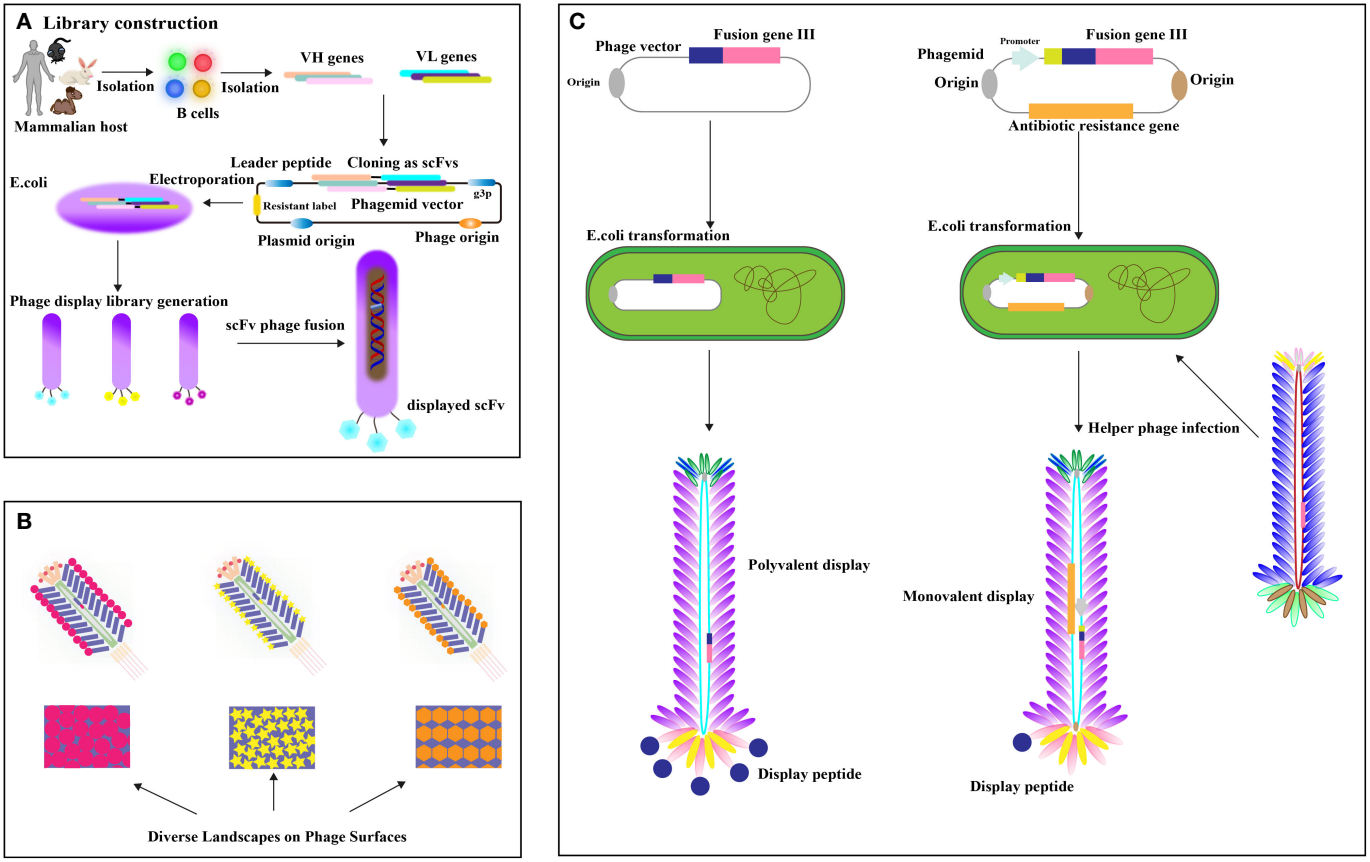
**(A)** Establishing the Phage Peptide Variant or Antibody Gene Library. Antibody libraries, fused with phage coat protein, are transformed into (E) coli. Enrichment of specific scFv-phage via target binding occurs, followed by removal of nonspecific or improperly folded scFvs through washing. Elution methods release specific scFv-phage fusions [adapted from (25)]. **(B)** Landscape Phage Concept: Illustrated is a distinct peptide “landscape” exhibited on the phage surface [adapted from (11)]. **(C)** Phage vector and selection: display valency is pivotal. Phage vectors carry recombinant genes for polyvalent display, while helper phages yield lower valence (typically pIII univalent) [adapted from (11)].

Phage display antibody (PDA) libraries created from immune donors are a direct method for isolating high-affinity antibodies against tumor-specific antigens, leveraging the antibody repertoire of cancer patients (20–22). This involves extracting mRNA from B lymphocytes, cloning it into phage coat protein genes like pIII, and expressing these in Escherichia coli to display antigen-binding domains (23, 24) (**Figure 2A**). Cancer patients frequently develop high-affinity antibodies due to tumors overexpressing or harboring mutated antigens (26). Utilizing immune phage display libraries from humans, researchers compile extensive antibody gene repertoires from cancer patients to isolate antibodies with specific binding capabilities (27). Studies include constructing scFv libraries from patient PBMCs, identifying high-affinity antibodies to EphA2 (28), and using cell panning to bind EGFR-affine fragments to esophageal cancer cells (29). Further developments involve producing nanobodies targeting HIF-1 and CD20/CD3 bispecific nanobodies from immunized camels, showing potential in cancer diagnostics and treatment (30–32). Camel-derived CD16a-specific antibodies conjugated with anti-CEA antibodies have effectively inhibited CEA-positive tumor growth (33), and nanobodies against EpCAM have significantly reduced MCF-4 cell proliferation (34). A VNAR antibody library from nurse sharks yielded antibodies binding to GPC3, HER2, and PD-1 (35), demonstrating phage display’s potential despite immunogenicity concerns (36). The phage display targets crucial cancer biomarkers and therapy targets like EGFR (37), its mutant EGFRvIII (38, 39). The Vascular Endothelial Growth Factor (VEGF), essential for angiogenesis, is targeted to inhibit tumor growth and metastasis (31), with phage display libraries from mice immunized against recombinant human VEGF (40). Landscape phage, defined as M13 phages with 5-mer phage peptides in all p8 protein copies, display thousands of ligands (41), providing a rich source of essential binding units (EBUs) for interacting with physiologically relevant proteins via short linear motifs (SLiMs) (42) (**Figure 2B**). Notable examples include Lei Han et al. (2018)’s high-affinity phage probe for prostate-specific antigen, applicable in ovarian cancer antigen detection (43), and V. Petrenko (2018)’s discussion on landscape phage’s role in nanobiotechnology, emphasizing their utility in cancer biomarker detection (41). The valency differences between phagemid and phage displays are significant (**Figure 2C**). Recombinant human antibody libraries, produced in bacterial or yeast hosts through recombinant DNA technology, mimic the human immune response, targeting diseases impartially using synthetic genes or unselected human B cells. Displayed on phages, yeast, or bacteria, antibodies are screened through biopanning against disease-specific targets and refined for high affinity and specificity. This approach generates antibodies effective against various diseases like cancer, autoimmune disorders, and infections, suitable when traditional immunization is impractical. Such technologies, which contribute to therapies like adalimumab, play a crucial role in advancing personalized medicine and improving treatment outcomes. Valency in phage and phagemid systems indicates the number of displayed peptides or antibodies. Phage systems like M13 often enable polyvalent displays, increasing binding strength through multivalent target interactions. In contrast, phagemid systems typically offer a monovalent display, with each particle showing a single antibody due to helper phage infection of plasmids with a phage origin of replication, allowing precise affinity measurements. Although phage systems are ideal for initial high-avidity screenings, phagemid systems are favored for their ease of genetic manipulation, accurate affinity assessment, and scalability, making them preferred for developing high-affinity therapeutic antibodies in advanced screenings.

Challenges include antigen heterogeneity and immunogenicity from non-human sources like camels and sharks. Future efforts should refine library construction and enhance screening for greater specificity and reduced immune reactions. Additionally, integrating disease-agnostic recombinant human antibody libraries could widen their application across various cancers. Exploring differences in valency between phage and phagemid systems is crucial, as phage systems facilitate high-throughput screening and phagemids provide precise affinity measurements essential for therapy. Advancing these technologies will require integrating both systems to address these challenges and improve cancer treatment outcomes.

### 2.3 From in situ to human-specific targeting

**Figure 3:**
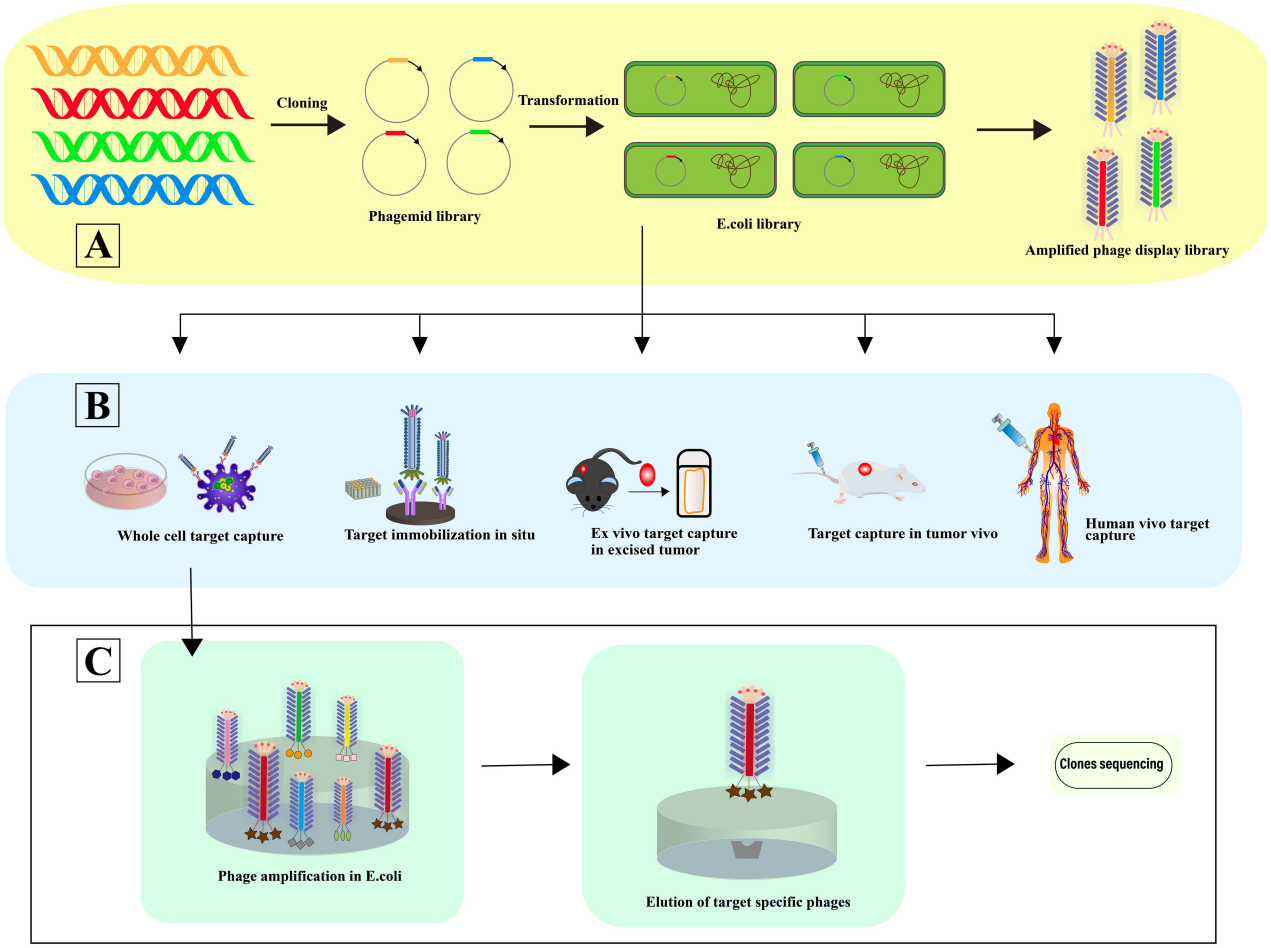
Phage selection. **(A)** In this process, a vast array of library variants is incorporated into phagemids, facilitating the transformation of (E) coli and subsequent phage rescue. The result is the creation of extensive phage libraries, which support the iterative biopanning process for the selection and isolation of phages that bind specifically to target antibodies. **(B)** The diagram illustrates Various Strategies for Harvesting High-Affinity Peptides Through Phage Display Screening: In situ screening involves the application of targets onto plates. Ex vivo screening is designated for isolating rare cells amidst heterogeneity. Screening using samples from human patients helps minimize species mismatches. *In vitro* screening is tailored to identify peptides specific to adherent cells, while organ-specific peptides are derived through *in vivo* biopanning and selection within living organisms [adapted from (50)]. **(C)** This is followed by further screening and sequencing of the identified phages, advancing towards the pinpointing of optimal target-specific ligands.

Phage selection involves a systematic four-step process: 1) Co-incubation with the target for selective binding; 2) Removal of non-specifically bound phages; 3) Elution of specifically bound phages; 4) Amplification of these phages for subsequent rounds of selection. This selection can occur in various contexts: In situ selection, a straightforward and commonly employed method, utilizes surfaces such as plates or beads for binding (44). *In vitro* cell selection is designed to identify phage-peptides specific to cell types, maintaining their biological activity, structural integrity, and receptor interactions, crucial for preserving their functional relevance (45). *In vivo* selection within animal models facilitates the isolation of phage-peptides that are specific to certain organs, providing insights under physiological conditions (46). Here, phage libraries are administered intravenously, allowing phages to circulate and bind organ-specifically. Phages are then harvested from targeted organs, with sequences identified post-homogenization (47). Ex vivo screening is particularly useful for identifying specific phage-peptides in rare cells within mixed populations, such as peripheral blood mononuclear cells (PBMCs) in hematologic malignancies (48). Utilizing human phage display libraries helps to minimize the variability in phage-peptide binding affinity that may arise due to interspecies differences (49), thereby enhancing the precision of cancer targeting in patient-specific therapies (50) (**Figure 3**).

Phage display challenges include inefficient step execution and slow clinical translation due to procedural inconsistencies and variable experiments, leading to unreliable peptide identification. Enhancements in phage selection could involve advanced molecular and computational tools to refine peptide specificity and functionality. High-throughput sequencing and machine learning could improve peptide insights, while better *in vivo* models might enhance translational potential. Utilizing human-specific phage libraries could diminish immunogenicity and improve outcomes, enhancing cancer targeting precision in personalized medicine. Adopting an integrated approach with human-specific libraries is crucial to advancing personalized therapies in phage selection.

## 3 Advancements and challenges in ovarian cancer immunotherapy

**Figure 4:**
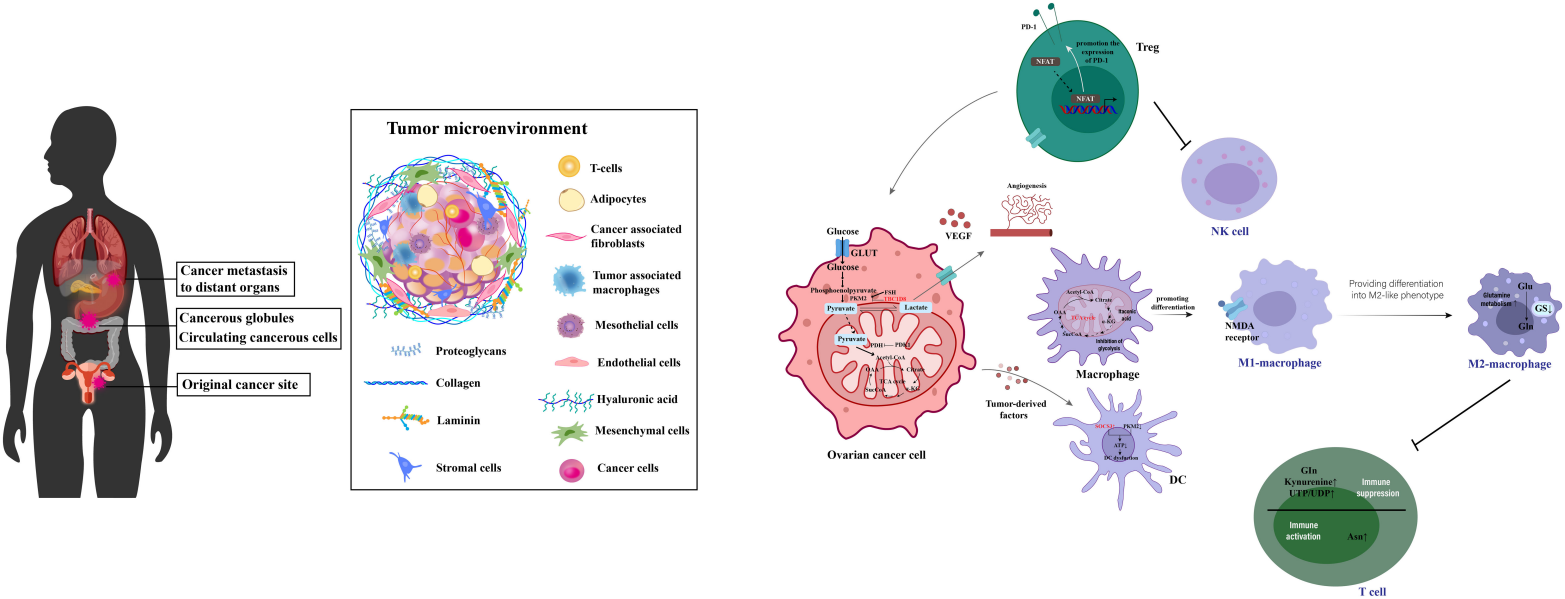
This illustration captures the process of ovarian cancer metastasis and the intricacies of its tumor microenvironment. It shows how ovarian cancer typically begins in the fallopian tube linings and spreads to the abdominal cavity. The diagram highlights the dynamic transitions between epithelial and mesenchymal states that are critical for the formation of secondary lesions. It further details the metabolic reprogramming within various cell types, including cancer and immune cells, contributing to a suppressive immune environment. This environment promotes tumor growth and progression by enhancing immunosuppression, facilitating robust cellular interactions, and enabling immune escape through a complex network of interconnected metabolites. [adapted from (57, 58)].

Ovarian cancer, a significant challenge within gynecologic oncology (51), is characterized by promising survival outcomes when identified at an early stage (52). Yet, the non-specific nature of its initial symptoms (53) frequently results in diagnoses during advanced stages (54). Survival rates for late-stage diagnoses fall under 20%, in stark contrast to the 90% survival rate associated with stage I detection (55, 56). Ovarian cancers mainly arise from the fallopian tube linings and metastasize to the abdominal cavity, where cells transition between epithelial and mesenchymal states to form complex secondary lesions with varied cell types and altered metabolism that supports rapid growth, as depicted in **Figure 4**. Despite ongoing research endeavors, the complexities of ovarian cancer detection and management persist, underscoring the imperative need for novel diagnostic and therapeutic strategies (59).

### 3.1 Surgical and chemoradiation therapies

Ovarian cancer treatment typically involves postoperative chemotherapy and surgery, especially important in early stages. Advanced-stage treatment generally consists of debulking surgery followed by adjuvant chemotherapy (60, 61). Patients unsuitable for optimal cytoreduction due to age or comorbidities might receive neoadjuvant chemotherapy and interval debulking surgery (62). The standard chemotherapy regimen often includes paclitaxel/carboplatin and pegylated liposomal doxorubicin/carboplatin (63), with olaparib recommended for remission patients with BRCA mutations post-initial platinum-based chemotherapy (± bevacizumab) (64). Despite initial responses, the high recurrence rate of ovarian cancer and platinum resistance complicate long-term management, with surgical complications and treatment variability demanding personalized care strategies to effectively address individual patient needs.

### 3.2 Utilization of targeted therapies and current challenges

In ovarian cancer treatment, targeted therapies like anti-angiogenic agents and PARP inhibitors offer significant hope (65). Bevacizumab is effective in reducing ascites and increasing progression-free survival initially (66), though its use is limited by high costs (67) and resistance (68). PARP inhibitors like Olaparib, Rucaparib, and Niraparib benefit ovarian cancer patients with BRCA1/2 mutations or homologous recombination deficiency (HRD) (69). Olaparib monotherapy yielded a 31.1% tumor response rate in platinum-resistant recurrent ovarian cancer (70), and Niraparib improved progression-free survival in late-stage patients (71). PARP inhibitors have been observed to enrich ovarian cancer stem cell populations marked by CD133 and CD117 *in vitro* and *in vivo* (72). ABBV-085 targeting LRRC15 has been effective in eliminating LRRC15-positive ovarian tumor cells in micrometastases and ascites (73). MFAP5 antibodies reduced fibrosis and enhanced chemosensitivity (74), while OMTX705 targeting FAPα with chemotherapy completely inhibited tumor growth and induced sustained regression in animal models (75). Research on the EZH2 inhibitor has focused on SCCOHT for ovarian cancer therapy (76), and the antibody-drug conjugate 3A5-MMAE targeting the MUC16-MSLN interaction significantly reduced ovarian cancer cell adhesion and invasion, demonstrating potent antitumor efficacy (77).

### 3.3 Applications and current challenges in immunotherapeutic approaches

**Figure 5:**
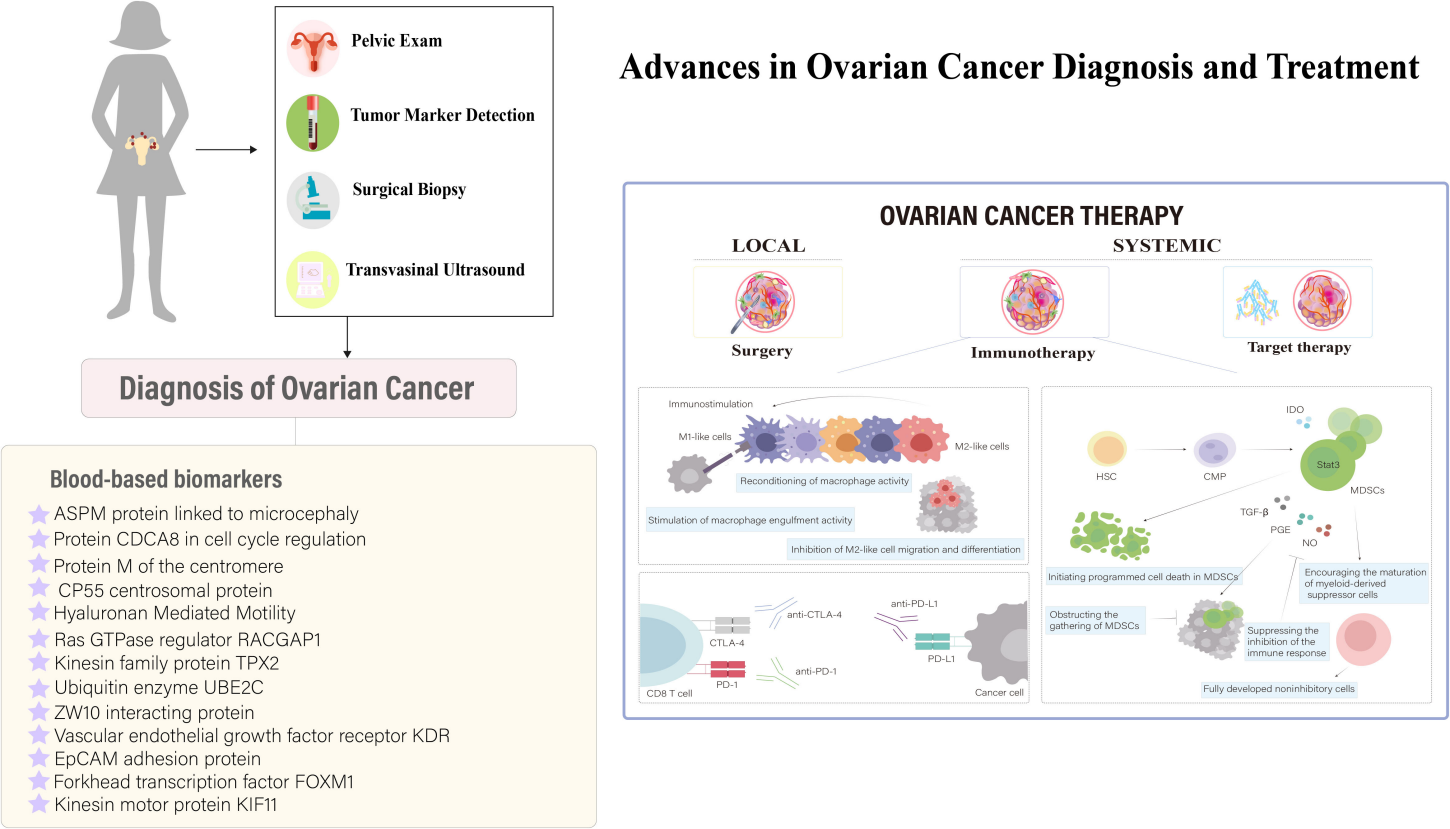
Innovations in Ovarian Cancer Immunodiagnostics and Therapeutic Strategies. This figure illustrates the critical role of serum biomarkers and nanosensors in diagnosing ovarian cancer, a condition typically managed through debulking surgery followed by systemic treatments like chemotherapy, targeted therapy, and immunotherapy. It highlights how conventional treatments modify the immune landscape, initially promoting immunosuppression for healing and subsequently enhancing immunostimulation to fight the disease. These dynamics underscore the importance of advancing immunotherapeutic strategies to maximize treatment effectiveness. [adapted from (78, 79)].

Recent studies on ovarian cancer have shown that serum biomarkers and nanosensors are crucial for diagnosis. Conventional treatments like debulking surgery and chemotherapy modify the immune response, first by increasing immunosuppression for healing, then boosting immunostimulation. This understanding is key to refining immunotherapies to enhance efficacy (**Figure 5**). In ovarian cancer treatment, immunotherapies like personalized vaccines using autologous dendritic cells and anti-PD-L1 antibodies are in clinical trials, showing potential to enhance adaptive immunity (80, 81). Genetically modified CAR-T cell therapy targeting tumor-associated markers like the folate receptor and mesothelin amplifies antitumor effects (82–84). Genetically engineered oncolytic viral therapies target ovarian tumors selectively, reducing non-tumor cell toxicity (85). Immunotherapy is increasingly integrated as a supplementary treatment, particularly against resistance to established therapies like bevacizumab and PARP inhibitors (86). Synergistic uses of αPD-1 and AMD3100 convert macrophages from M2 to M1, enhancing antitumor responses (87), while PI3K pathway inhibitors combined with bevacizumab (88), and VEGF antibodies with tumor vaccines enhance vaccine efficacy by modulating CD4+ CTL populations (89). Combining PARP inhibitors with PD-L1 inhibitors shows promise in recurrent ovarian cancer treatment by modulating the immune response (90), highlighting the disease’s heterogeneity and propensity for recurrence and metastasis (56).

## 4 Phage display applications in ovarian cancer therapy and diagnosis

### 4.1 Specific targeting phage-peptide identification for ovarian cancer

**Figure 6:**
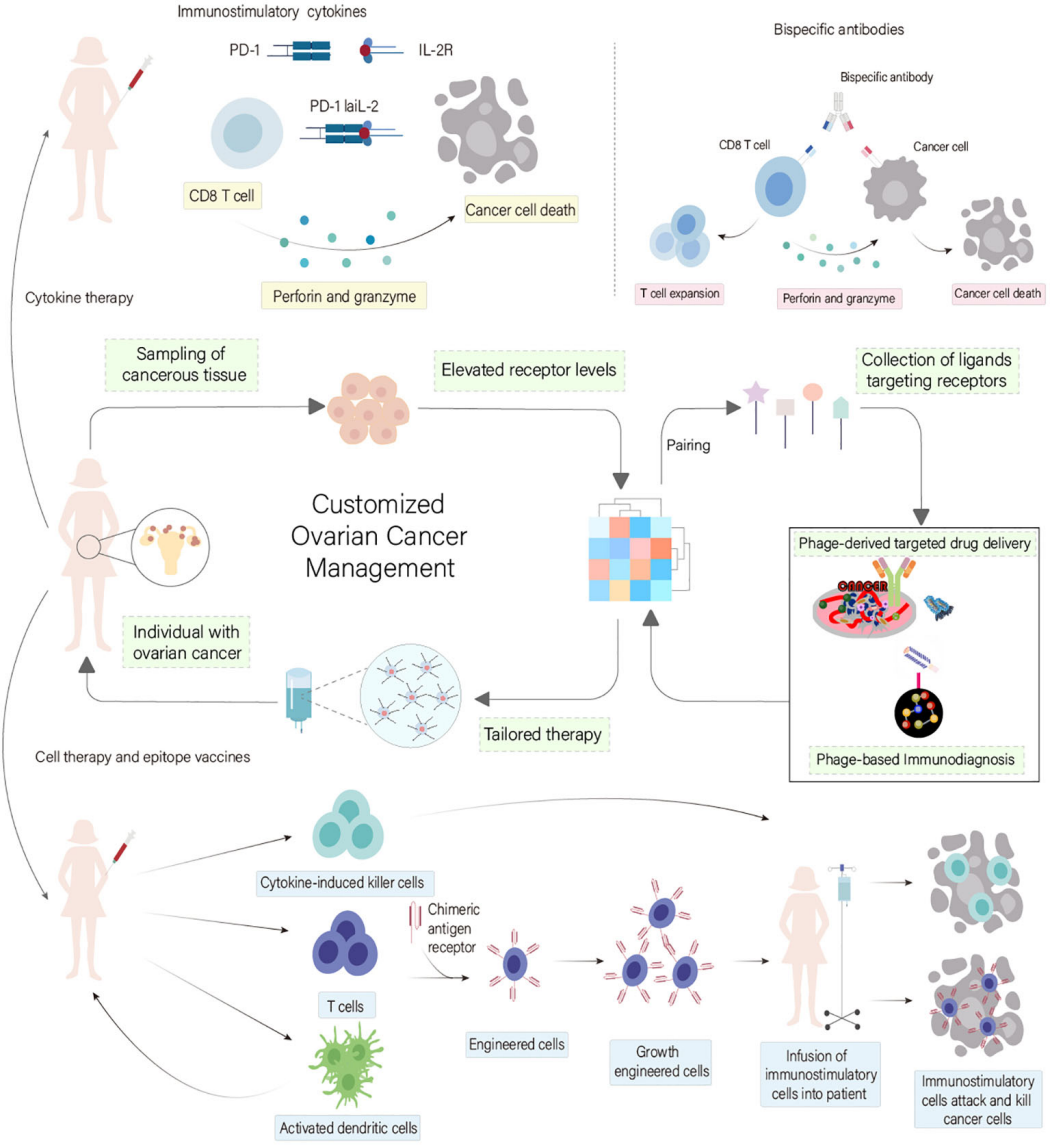
Phage Display Technology in Ovarian Cancer Treatment Customization. This diagram underscores the transformative role of phage display technology in ovarian cancer immunodiagnostics and therapy. It showcases how phage display facilitates the identification of specific ligands that target ovarian cancer cells, thus paving the way for innovative diagnostic and therapeutic approaches. This advancement enhances drug specificity and minimizes side effects, promising to revolutionize treatment modalities for ovarian cancer. [adapted from (57, 79)].

Phage display emerges as a transformative tool in ovarian cancer research, offering the capability to identify ligands that precisely target cancer cells, potentially ushering in new paradigms in diagnosis and treatment by enhancing drug specificity and reducing side effects (91) (**Figure 6**). Zhou et al. successfully leveraged phage display to pinpoint a unique phage-peptide, SWQIGGN, demonstrating its capacity to bind and impede the functions of ovarian cancer cells both *in vitro* and *in vivo*. This phage-peptide exhibited selective affinity for cancer cells, significantly curtailing their proliferation, migration, invasion, and adhesion. Moreover, it notably inhibited tumor growth and metastasis in animal models, setting the stage for further exploration into SWQIGGN’s clinical utility in ovarian cancer treatment (92). Wang et al. addressed the critical demand for phage-peptides with high specificity as targeting agents for the early detection of ovarian cancer. They proposed the use of phage display technology to identify phage-peptides uniquely associated with cancer cells. By integrating a novel microfluidic system, they streamlined the screening process, enhancing the efficiency and reducing the laborious nature of traditional methods. The identified phage-peptides displayed remarkable affinity and specificity to ovarian cancer cells, indicating its potential in targeted immunodiagnostics. This method’s increased efficiency, minimal sample requirements, and accelerated screening process hold promise for advancing ovarian cancer theranostics (93). Davidson et al. explored the application of phage display in biomaterials engineering, aiming to discover phage-peptides with high affinity for specific targets. These phage-peptides can be engineered to functionalize material surfaces, precisely guiding interactions within the biological milieu. By employing targeting phage-peptides, materials can be designed to selectively interact with proteins, cells, or tissues. Moreover, functional phage-peptides are capable of not just binding but also influencing target activities. The integration of targeting and functional phage-peptides paves the way for the development of dual-functional phage-peptides, enabling the bridging of distinct targets or the modulation of specific protein or cell functions (94), illustrating the versatile potential of phage display in advancing ovarian cancer research and treatment modalities.

### 4.2 Phage-derived specific peptides for targeted drug delivery

Conjugating therapeutic agents to targeting phage-peptides or antibodies identified through phage display can directly target cancer cells, substantially reducing off-target effects and sparing healthy tissues. This technique enhances the potential for precise drug delivery, significantly advancing the pursuit of personalized medicine by allowing for the screening of novel targeting ligands tailored to individual cancer profiles. As innovations progress, phage display-mediated targeted drug delivery is set to transform cancer therapy, heralding a new era of efficient and customized treatments (95). Ma et al. unveiled a promising phage-peptide, WSGPGVWGASVK, identified via phage display for targeting human ovarian cancer, potentially revolutionizing cancer therapy. This phage-peptide ‘s *in vivo* application demonstrated selective tumor site accumulation post intravenous administration, minimizing normal organ distribution. Immunostaining revealed PC3–1 phage clone’s affinity for tumor cells and vasculature, ensuring comprehensive tumor binding. *In vitro* assessments further confirmed the phage-peptide ‘s tumor cell and angiogenic endothelial cell binding and internalization capabilities, with a dissociation constant (Kd) of 5.43 ± 0.4 μM, underscoring its therapeutic delivery potential (96). An innovative active targeting liposome system for ovarian cancer, developed using phage display, incorporates the ovarian-specific ligand WSGFPGVWGASVK (WSG) on thermosensitive phospholipids, forming WSG-modified thermosensitive liposomes (WSG-Lipo). These liposomes showcase enhanced drug release at 42°C, targeting SKOV-3 ovarian cancer cells and suppressing their activity *in vitro*. This strategy hints at the WSG-Lipo’s potential to refine thermosensitive liposome specificity, promising advances in combined chemotherapy and thermotherapy for ovarian cancer (97). Addressing the challenge of treating clear cell carcinoma, a notably aggressive and chemoresistant epithelial ovarian cancer subtype, researchers utilized phage display to isolate specific phage-peptides (OC-6 and OC-26) targeting this cancer form. Peptide-conjugated nanoparticles significantly improved tumor cell uptake and drug delivery internally, compared to non-targeted versions. Moreover, the targeted liposomal doxorubicin formulation achieved superior tumor suppression, indicating that phage display-derived subtype-specific therapies could offer more effective, targeted treatment options for varying epithelial ovarian cancer subtypes (98).

### 4.3 Imaging applications for ovarian cancer

Screening phage libraries has emerged as a pivotal method for identifying phage-peptides or ligands that target molecules uniquely expressed on ovarian cancer cells, facilitating early disease detection and precise diagnostics (99). Engineered phage-displayed peptides can be conjugated with imaging agents, such as fluorescent labels or radioactive markers, allowing for the direct visualization and mapping of ovarian cancer cells and lesions. Moreover, this technology is instrumental in creating innovative biosensors capable of detecting ovarian cancer biomarkers in bodily fluids, offering a minimally invasive, highly sensitive modality for ongoing disease surveillance. Wang et al. employed phage display to isolate NPMIRRQ, a phage-peptide that exhibits selective affinity towards HO-8910 ovarian cancer cells, demonstrating its utility as a diagnostic tool through specific ovarian cancer cell and tissue binding, potentially advancing the identification of reliable biomarkers for the disease’s early detection and management (100). Xing et al. discovered a phage-peptide, C7, via phage display, showcasing targeted binding to the folate receptor alpha (FRα), predominantly expressed in ovarian cancer cells. C7’s tumor-targeting capabilities were confirmed in *in vivo* studies, with structural modeling elucidating its interaction with FRα, underscoring C7’s therapeutic and diagnostic potential by leveraging FRα’s restricted tumor expression profile in ovarian cancer (101). Further, two phage clones, pJ18 and pJ24, along with their phage-peptides, J18 and J24, demonstrated enhanced affinity for SKOV-3 ovarian cancer cells relative to controls. AF680-labeled phage particles underwent biodistribution analysis, revealing tumor accumulation and retention, with optical imaging pinpointing SKOV-3 tumors post 2 and 4 hours. Particularly, pJ18 exhibited promising tumor-to-background contrast, suggesting its suitability for ovarian cancer detection and imaging (102). A dual-stage phage display technique was utilized to select phage-peptide J18 for its specificity to ovarian cancer cells. Radiolabeling J18 with (11) In and assessing its imaging prowess via SPECT in SKOV-3 xenografted mice, revealed efficient tumor localization with minimal nonspecific binding, indicating J18’s significant potential as a nuclear imaging agent for ovarian cancer diagnostics and treatment (103). Faintuch et al. investigated the NGR phage-peptide, identified through phage display, for radiolabeling with technetium-99m across different tumor models, noting similar uptake in ovarian and lung tumors. Biodistribution studies indicated renal clearance with higher ovarian tumor cell uptake, suggesting enhancements in tumor targeting and imaging by potentially combining NGR with RGD into a heterodimer (104). Babeker et al. reported on generating fully human antibodies against the MUC16 cancer biomarker via phage display. The derived antibody, M16Ab, was modified with p-SCN-Bn-DFO and labeled with 89Zr for immuno-PET imaging. Subsequent *in vitro* and *in vivo* assessments showcased specific, high-affinity MUC16 binding, especially in ovarian and pancreatic cancer models. Pharmacokinetic analysis revealed favorable attributes for the non-invasive imaging of these cancers (105), illustrating the broad and impactful applications of phage display in enhancing ovarian cancer diagnostics and targeted therapy.

### 4.4 Phage-based gene therapy for ovarian cancer

Yu et al. elucidated the utility of phage display in discovering anti-HE4 nanobodies, notably neutralizing the human epididymis protein 4 (HE4) and thereby attenuating the viability of epithelial ovarian cancer (EOC) cells while augmenting their responsiveness to cisplatin. This research underscores the autocrine pro-survival function of HE4 in EOC cells and positions the anti-HE4 nanobody, particularly 1G8, as an efficacious candidate to bolster cisplatin chemotherapy in EOC management (106). Huang et al. probed the fibroblast growth factor-2 (FGF2)’s contribution to ovarian cancer advancement and its therapeutic targeting viability. Elevated FGF2 levels in ovarian tumors were inversely related to patient survival, highlighting its prognostic significance. A heptapeptide-derived phage-peptide showcased inhibitory actions on FGF2-mediated proliferation, migration, and invasion in p53-null epithelial ovarian cancer cells, disrupting cell cycle progression, cyclin D1 upregulation, and MAPK/Akt pathway activation. Additionally, it showed promise in mitigating FGF2-driven doxorubicin resistance by downregulating antiapoptotic proteins and neutralizing FGF2’s antiapoptotic influence (107). Pu et al.’s work focused on devising targeted anti-metastatic therapies, pinpointing a phage clone pc3–1 that exhibited high affinity and specificity to SK-OV-3 cells via the phage-peptide WSGPGVWGASVK. This clone and its corresponding phage-peptide significantly hampered SK-OV-3 cell adhesion to the extracellular matrix and endothelial monolayers, also curtailing invasion (108). Chen et al. deployed a human phage display library to identify a potent anti-MSLN single-chain Fv antibody, facilitating the creation of a second-generation anti-MSLN CAR-T cell therapy. These CAR-T cells proved effective *in vitro*, eliminating ovarian tumor cells and suppressing MSLN-positive tumor growth, corroborated by elevated cytokine production. *In vivo* assays further confirmed their therapeutic potential against ovarian cancer xenografts (109). Qiao et al. aimed for enhanced affinity antibodies using phage display, employing 3D complex structural modeling of the HER2-antibody (MIL5) interface to guide the synthesis of a site-directed mutagenesis library. This approach yielded a higher affinity single-chain antibody (M5scFv_ph), maintaining the epitope specificity of its precursor and demonstrating comparable tumor-suppressive efficacy in ovarian carcinoma xenograft models (110). Bortot et al. explored M13 bacteriophage as a precision vector for photodynamic therapy (PDT) in ovarian cancer. By engineering M13 phage to present an EGFR-binding phage-peptide and coupling it with chlorin e6 (Ce6), they generated M13r-Ce6, which produced reactive oxygen species (ROS) upon irradiation, effectively eradicating EGFR-positive ovarian cancer cells. This modification ensured superior cellular uptake and mitochondrial localization, highlighting its potential to induce autophagy and augment PDT efficacy in ovarian cancer (111). **Table 2** provides a comprehensive overview of ovarian cancer-targeting phage-peptides identified through phage display, underscoring a decade of significant advancements in targeted cancer therapy research.

**Table 2:** Exploring Ovarian Cancer Targeting Peptides through Decade-long Phage Display Screening.

peptide sequence	phage clone	Target	Year	Application	Reference
SVSVGMKPSPRP	Z3	SKOV3 cells	2011	Diagnostics	(112)
GD3A10		glycosaminoglycans	2014	Diagnostics	(113)
OSTP		A2780 mice	2015	Diagnostics	(114)
GD3A11		Chondroitin sulfate (CS)	2015	Diagnostics	(115)
NPMIRRQ	P2	HO-8910 cells	2016	Diagnostics	(100)
CREB3		Igs	2019	Diagnostics	(116)
	S36	SKOV3	2021	Diagnostics	(117)
M16Ab		MUC16	2022	Diagnostics	(105)
WSGPGVWGASVK	pc3-1	SKOV3 mice	2013	Therapy	(96)
OR2H1 scFv		OR2H1	2022	Therapy	(118)
	A7	HB-EGF	2021	Therapy	(119)
OCSP-6 and OCSP-26	OC-6&OC-26	OC-3 cells	2015	Therapy	(98)
no. 7 and no. 29		HB-EGF	2019	Therapy	(120)
SWQIGGN		HO8910 cells	2015	Therapy	(92)
P2		FGF2	2019	Therapy	(107)
C7		FRα	2018	Therapy	(101)
8G2/8G3		HE4	2022	Therapy	(106)
anti-MSLN scFv		MSLN-His tag protein	2023	Therapy	(109)

## 5 Future directions and challenges

### 5.1 Advances and applications in obtaining targeted phage-peptides

Utilizing a phenotypic library screening strategy enables the identification of ovarian cancer-specific surface markers in a non-biased fashion, capturing physiologically pertinent targets. The application of single-domain antibody libraries, notably VHH libraries, facilitates the unveiling of targets with diverse expression profiles. This method’s adaptability lends itself well to various detection and therapeutic modalities. Notably, Her2 and BCAM have been pinpointed as promising candidates for targeted ovarian cancer therapy. Her2, in particular, merits further investigation within specific ovarian cancer subsets. BCAM, distinguished by its pronounced expression in high-grade serous ovarian carcinoma (HGSOC) and minimal presence in normal tissue, emerges as a compelling target for precision therapy endeavors. Moreover, the integration of phage display-derived antibodies with chemotherapy agents, such as paclitaxel, suggests a pathway to enhancing treatment outcomes while mitigating additional toxicity. The principal benefits of employing phage display for targeted phage-peptide acquisition include its efficiency and cost-effectiveness (121).

### 5.2 Current limitations and obstacles

In phage display systems, phage-peptides are showcased by coupling them with the coat proteins of filamentous bacteriophages. Yet, the diminutive size of these phages imposes limitations on the magnitude of proteins that can be displayed (122). Short phage-peptides, due to their linear and compact nature, often demonstrate restrained affinity, which can hamper their efficacy in target binding. This limitation poses potential obstacles in leveraging these phage-peptides as therapeutic agents (50). Additionally, the complex lifecycle of bacteriophages introduces challenges in showcasing post-translationally modified proteins or phage-peptides, such as those undergoing phosphorylation and glycosylation, on the phage surface. These modifications are crucial for protein folding, interactions, and signal transduction, thereby limiting the widespread application of phage display in these essential functions (123). The effectiveness of a bacteriophage display library, characterized by the variety and representation of displayed proteins or phage-peptides, plays a critical role in its practical utility. Ongoing investigations in this field are poised to explore novel avenues in science and medicine, potentially fostering breakthroughs in ovarian cancer treatment and other areas. Phage display, a leading platform for antibody discovery, is robust and excels in quickly screening extensive libraries for high-affinity binders. However, it struggles with post-translational modifications and complex antibody structures. Integrating phage display with yeast and mammalian technologies helps overcome these limits. Yeast display uses eukaryotic systems for accurate protein folding and modifications, while mammalian display presents antibodies on mammalian cells, ensuring human-like modifications critical for therapeutic use. This integration enhances antibody properties like stability and pharmacokinetics, and facilitates the discovery of therapeutically effective antibodies. By merging these technologies, researchers can produce superior antibodies more suited for therapeutic use, speeding the development of advanced antibody therapies.

### 5.3 Enhancing the phage display platform in therapy

Phage display technology offers a versatile toolkit for a broad spectrum of applications, from material science to the development of therapeutic agents (124). This method boasts several advantages over alternative non-phage systems, such as facilitating the discovery of antibodies in nonphysiological settings, identifying pH-sensitive antibodies with distinct binding characteristics, and generating recombinant antibodies against potent toxins. Despite its successes, the technology faces hurdles, including the presentation of hydrophobic phage-peptides on the phage surface and the propensity for recombinant proteins to aggregate. Advancements in phage display techniques are sought to address these challenges, with the investigation of thermophilic bacteriophages presenting promising avenues (125). The awarding of the Nobel Prize in Chemistry in 2018 to phage display technology underscores its pivotal role and achievements in identifying high-affinity phage-peptides from complex libraries. Within the realm of ovarian cancer therapy, this review explores the advancements in phage display methodologies aimed at isolating cancer-specific ligands through varied screening processes. These developments highlight the technology’s potential to refine targeted therapeutic strategies in clinical settings.

## 6 Conclusion

Ovarian cancer represents a significant contributor to female mortality worldwide, yet the availability of specific and high-affinity agents for its early detection remains scarce. Through the identification of phage-peptides that bind selectively and strongly to ovarian cancer cells, phage display technology facilitates the swift isolation and recognition of these cells, enhancing the speed and efficiency of immunodiagnostics. This method also holds promise for utilizing phage-peptides as precision targeting agents in ovarian cancer treatment. Furthermore, phage display aids in uncovering novel oncogenic targets, such as BCAM, offering promising avenues for targeted interventions in high-grade serous ovarian cancers. This technology establishes a versatile foundation for the identification of cancer-specific targets and antibodies, contributing significantly to the advancement of targeted therapy strategies.

## Author contributions

YL: Conceptualization, Data curation, Formal analysis, Funding acquisition, Investigation, Methodology, Project administration, Resources, Software, Supervision, Validation, Visualization, Writing – original draft, Writing – review & editing. X-ML: Methodology, Visualization, Software, Writing – original draft. K-DY: Conceptualization, Data curation, Investigation, Software, Writing – original draft. W-HT: Investigation, Software, Supervision, Writing – review & editing.
